# Density of dopaminergic fibres in the prefrontal cortex of gerbils is sensitive to aging

**DOI:** 10.1186/1744-9081-3-14

**Published:** 2007-03-12

**Authors:** Susanne Brummelte, Gertraud Teuchert-Noodt

**Affiliations:** 1 Department of Neuroanatomy, Faculty of Biology, University of Bielefeld, Universitätsstr. 25, D-33615 Bielefeld, Germany

## Abstract

Mesencephalic dopamine (DA) projections are essential for cognitive and behavioral functions and believed to play a critical role during development and aging. The dopaminergic afferents of the rodent prefrontal cortex (PFC) show an extremely prolonged maturation which is very sensitive to epigenetic challenges. However, less is known about the long-term maturation and aging of these DA axons. Therefore, immunohistochemically stained DA fibres were quantitatively examined in the PFC of the Mongolian gerbil (*Meriones unguiculatus*) ranging from 6 to 24 months of age. Results show a decrease in DA fibre densities in the superficial layers of the PFC in 24 month old animals compared to 6 and 12 months.

## Findings

Dopamine (DA) has frequently been associated with age-related changes and neurodegenerative diseases such as Parkinson. In particular, striatal alterations have been in the focus of many investigations, as these are assumed to contribute to observed cognitive and motor dysfunction in elderly people or Parkinson patients [1].

However, recent studies also suggest age-related DA changes in extrastriatal brain regions. Mirura and colleagues [2] observed that the level and turnover of monoamines and their metabolites were reduced in several brain regions as e.g. the prefrontal cortex (PFC), the amygdala, nucleus accumbens and hippocampus of 18 months old rats compared to young animals. For humans, it has been shown that the DA synthesis is reduced with age in several extrastriatal regions, including the dorsolateral prefrontal and anterior cingulate cortex [3]. In addition, an age-related decline in D2 receptors was also found in various extrastriatal areas of healthy volunteers suggesting an association with normal aging processes [4]. In fact, it appears that the declines in D1 and D2 receptor binding might even be faster or more pronounced in the frontal cortices compared to striatal or thalamic regions [4-6]. This is in line with other studies reporting a greater loss of DA from the PFC compared to motor areas in aged monkeys [7,8], which underlines the importance of dopaminergic function during aging in this area.

So far, most studies have focused on the metabolic function of the dopaminergic system during aging, but less research has been done concerning neuroanatomical alterations. Our laboratory could recently show, that the dopaminergic fibre densities of the nucleus accumbens, the amygdala and the entorhinal cortex show no age-related changes in 24 month old gerbils (*Meriones unguiculatus*) compared to young animals [9,10]. However, as the PFC has been frequently associated with an age-related decline in cognitive function, this study was conducted to check for alterations in the dopaminergic fibre density in this particularly vulnerable area. We focused on the prelimbic cortex (PrL) and the infralimbic cortex (IL), since the first area is known to be involved in higher-order cognitive functions, while the second is a visceromotor cortex, with both subregions exhibiting individual as well as overlapping connectivity patterns [11,12].

All experimental procedures were approved by the appropriate committee for animal care in accordance with the European Communities Council Directive. Gerbils were chosen due to their wild-type like behavioural and neuronal repertoire, as they have not been so intensively domesticated compared to rats or mice [13]. A total of 33 male Mongolian gerbils were used in this study (6 Mon n = 8; 12 Mon n = 5; 18 Mon n = 11; 24 Mon n = 9). Animal rearing and keeping conditions as well as the DA staining procedure have been described elsewhere [9].

Prefrontal DA fibre densities were measured in four consecutive coronal slices of the PFC. Fibre fragments in the upper layers were visualised in standard test fields in the PrL and in the IL, using a bright-field microscope (BX61, Olympus, Hamburg, Germany) and a digital camera for microscopy (ColorView II, SIS, Münster, Germany) at 400-fold magnification. Fibres were quantified by software for image analysis (KS300, Jenoptik, Jena, Germany). For details of the quantification see [9]. The fibre area was calculated as a percentage of the reference area. All measurements were done by an experimenter blind to the coding of the samples.

Measurements were computed as arithmetic means by-case and by-group ± S.E.M. and a two-way analysis of variance (ANOVA) with age (4 levels) and area (2 levels) as independent variables and the dopaminergic fibre density as the dependent variable was used to check for statistical significance between groups followed by LSD post-hoc test for multiple comparisons. Statistical analysis was computed with Statistica 6 (StatSoft, Tulsa, USA). The levels of significance were set at * p < 0.05, ** p < 0.01 and *** p < 0.001.

**Figure 1:**
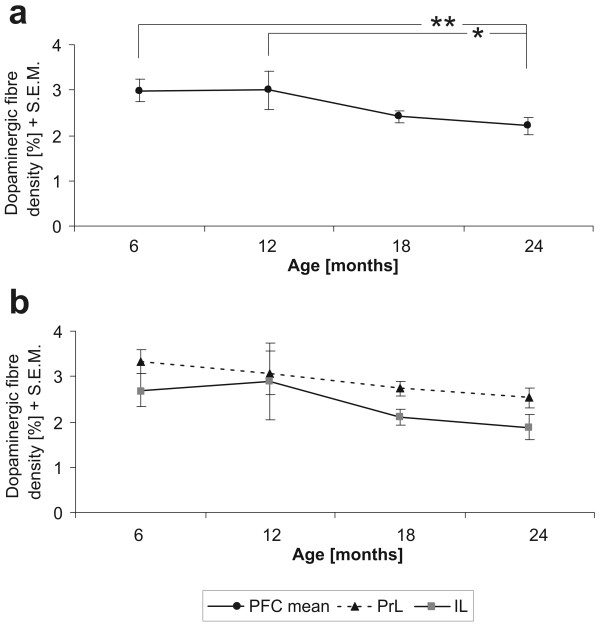
Development of dopaminergic fibre densities in the prefrontal cortex (a). There is a significant decline in the density in 24 months old animals compared to 12 months and 6 months old gerbils. Picture (b) shows the developmental patterns of the prelimbic (PrL) and infralimbic cortex (IL) separately.

Statistical analysis revealed a significant effect of age (F(3,56) = 3.47; p = .022) and area (F(1,56) = 5.53; p = .022), but no interaction effect (F(3,56) = .184; p = .907). The PrL cortex showed a dense innervation of DA fibres, which was according to a Fisher LSD post-hoc test significantly lower in the IL (p = .008). The post-hoc test further revealed a significant age-related decrease in DA fibre density in the superficial layers of the PFC between 12 month and 24 month old animals (-26%; p = .025), with the significance being even more prominent compared to 6 month old gerbils (-26%; p = .0098) (Fig. 1).

Thus, we here present evidence for age-related anatomical alterations in the dopaminergic innervation pattern of the gerbil PFC. The decrease in DA fibre densities we found in the superficial layers of the PFC is in line with other observations of age-related alterations in the dopaminergic system. For instance, it has been shown, that the stress-related increase of dopamine diminishes with age as well as the dopamine transporter densities [14,15]. Thus, it has been assumed that the dopamine depletion of the PFC might contribute essentially to age-related cognitive declines [16].

Remarkably, previous studies in the gerbil could not detect a decline in DA fibre densities in other brain areas than the PFC in old animals compared to adult ones [9,10]. The different vulnerability of DA fibres in distinct areas might be related to varying maturation patterns of the DA fibres. The dopaminergic fibre densities of the PFC reveal a prolonged maturation until early adulthood [17,18] while the innervation patterns of other areas mature relatively early. This ongoing increase in fibre density has been assumed to be associated with a continuously high plasticity within the PFC, but also with a high vulnerability concerning external influences [19]. The observed decline in DA fibres in the gerbil PFC of 24 month-old animals reflects an age-related disturbance in the DA system, which might also be related to the high plasticity in this area, and possibly result from reactive or adaptive processes following other physiological changes. Interestingly, an adult pharmacological challenge only induced significant long-term effects of the dopaminergic fibre densities in the shell region of the nucleus accumbens, but not in the PFC [20]. However, the present results are in line with observations from Ishida and co-workers [21] who found an early reduction of noradrenergic innervations in the frontal cortex of aging rats. In addition, it has been shown, that aging can change the interaction of different transmitters in the brain [22]. As the PFC is known to have several controlling connection over other brain systems and hence can essentially influence behavioural and cognitive functions, it seems likely that a disturbance within this superior cortex division might have extensive and far-reaching consequences for other areas and their function.

## Competing interests

The author(s) declare that they have no competing interests.

## Authors' contributions

SB contributed to the bench work, analysis and interpretation of the data and the drafting and revision of the manuscript

GT contributed to the design of the study and the critical reviewing of the manuscript.
